# Cognitive Deficits and White Matter Alterations in Highly Trained Scuba Divers

**DOI:** 10.3389/fpsyg.2019.02376

**Published:** 2019-10-22

**Authors:** Marinella Coco, Andrea Buscemi, Valentina Perciavalle, Tiziana Maci, Gianluca Galvano, Antonio M. F. Scavone, Vincenzo Perciavalle, Donatella Di Corrado

**Affiliations:** ^1^Department of Biomedical and Biotechnological Sciences, University of Catania, Catania, Italy; ^2^Study Center of Italian Osteopathy, Horus Social Cooperative, Catania-Ragusa, Italy; ^3^Department of Educational Sciences, University of Catania, Catania, Italy; ^4^Independent Researcher, Catania, Italy; ^5^U.O.C. Diagnostic Imaging, Interventional Radiology and Neuroradiology, Garibaldi Hospital, Catania, Italy; ^6^Faculty of Human and Society Sciences, Kore University of Enna, Enna, Italy

**Keywords:** scuba diving, white matter, Fractional anisotropy, neuropsychological testing, sport

## Abstract

Nitrogen gas (N2), present in the normal atmospheric air, is a potential source of risk for scuba divers. It seems probable that myelin can represent, in hyperbaric conditions, a preferential site for the accumulation of N2 in central nervous system (CNS). The purpose of this study is to verify whether the practice of the scuba diving is capable to determine a damage of the brain white matter (WM) in a dose dependent manner and, consequently, possible deficiency of their cognitive abilities. For this purpose, 54 professional scuba divers (35 men and 19 women), with at least 2,000 dives in their careers, were studied. Possible alterations of brain WM were evaluated in terms of Fractional anisotropy (FA) by using Diffusion Tensor Imaging, whereas possible cognitive impairments were verified by means of neuropsychological testing, by studying: (1) General mental capability (2) Executive functioning; (3) Visuospatial construction such as Rey Complex Figure; (4) Attention and orientation: (5) Selective attention capacity and processing speed ability; (6) Memory. The results showed alteration of the WM in terms of changes in FA; these alterations, statistically significant but quantitatively quite modest, were mainly observed in the WM of the anterior part of the brain, whereas no differences were observed between left and right hemisphere. The alterations of the WM were associated with changes, also in this case statistically significant but quantitatively quite modest, of the cognitive functions, in particular of those dependent on the prefrontal cortex, such as attention and memory function. The present study leads to the conclusion that repeated dives, even performed in compliance with the current decompression tables, can progressively lead in the CNS to the formation of micro-lesions in the myelin sheet capable of altering the functioning of the neuron.

## Introduction

Nitrogen gas (N2), present in the normal atmospheric air, is a potential source of risk for scuba divers for two different reasons: during the descent may induce nitrogen narcosis and during the ascent may cause decompression sickness (DCS).

Individuals exposed to increasing N2 pressures exhibit, already at 0.3 MPa, spatial disorientation, altered judgment and impaired neuromuscular coordination (Bennett, 1993), up to the loss of consciousness when the pressure of N2 reaches 1 MPa, a condition related to nitrogen narcosis (Edmonds et al., 1981). The narcotic effect is dependent on to the increase in the partial pressure of N2; the gas in fact, with the increase in pressure, would dissolve in the cell membrane of the neuron (Behnke et al., 1935). In this way, the increased presence of N2 in the cell membrane on the one hand would modify its biophysical characteristics and, on the other hand, the binding of N2 to membrane proteins, would vary their activity (Franks and Lieb, 1994).

It is known that DCS occurs when N2 dissolved in the tissues is released too quickly during decompression, leading to the formation of bubbles that can cause damage to various organs (see Doolette and Mitchell, 2001). The relationship between N2 and adipose tissue is of particular attention in hyperbaric conditions as N2 is five times more soluble in lipids than in water (Weathersby and Homer, 1980; Langø et al., 1996). According to this point of view, the adipose tissue would act as a reservoir for N2, able to accumulate it during the compression phase and then quickly release it during decompression; in fact, it has been shown that the risk of DCS is greater in obese subjects (Carturan et al., 2002).

In the human body lipids are involved in two essential functions: in the form of triglycerides, they are an excellent substrate to be oxidized for producing energy and they are an essential component in the structure of cell membranes. Since the central nervous system (CNS) does not contain triglycerides, brain lipids have been studied to identify roles different from that of energy substrate. Actually, in addition to structural tasks in cell membranes, as myelin, they act as bioMessengers and intervene in cellular signal transduction processes (see Agranoff et al., 1999).

Myelin is a lipid-rich substance that is produced by specialized glial cells, that in the CNS are the oligodendrocytes and in the peripheral nervous system the Schwann cells (see Nave and Werner, 2014). At the level of the axons, the myelin acts as an electrical insulator, favoring the propagation of the nerve impulse at high speed from one node of Ranvier to the other (Waxman, 1980). Lipids represent about 70% of the dry weight of myelin (Norton and Poduslo, 1973), a value that is about twice that measured in the other cell membranes (Chrast et al., 2011).

From a logical point of view, it seems probable that myelin can represent, in hyperbaric conditions, a preferential site for the accumulation of N2 in CNS. However, there are no studies about the possible alterations of myelin in scuba divers, in particular in those that, for professional reasons, perform thousands of dives. It is conceivable, in fact, that a continuous exposure to conditions of high partial pressures of N2 could lead, during the decompression phase, to the formation within the myelin of microbubbles which could compromise the functioning of white matter (WM) in the CNS.

The aim of this study was to verify whether the practice of the scuba diving is capable to determine a damage of the brain WM in a dose dependent manner and, consequently, possible deficiency of their cognitive abilities. Recent literature has documented effects of diving on cognitive functions (Kowalski et al., 2011; Ergen et al., 2017; Howle et al., 2017; Bosco et al., 2018; Verratti et al., 2019). Some of these studies have reported decreased mental flexibility (Cordes et al., 2000), as well as lower verbal memory and intelligence (Hemelryck et al., 2014), sustained attention in divers reporting memory and concentration loss (Taylor et al., 2006), and decrease average response speed (Pourhashemi et al., 2016). Further, we expected that a high number of dives (at least 2,000) with compressed air is able to alter the quality of brain WM, evaluated by using diffusion tensor imaging (DTI), determining cognitive impairments.

## Materials and Methods

### Participants and Procedure

Fifty-four professional scuba divers (35 men and 19 women) in the age range from 28 to 58 years (M_*age*_ = 40.1 years, SD = 6.08) were recruited for the study. For scuba divers, inclusion criteria were to have performed at least 2,000 scuba dives, with an average of at least 100 dives a year, proven by their personal dive log. Controls were represented by 54 individuals (35 men and 19 women) having the same mean age of divers (mean value 39.7 years ± 7.22 SD). Table 1 summarizes the main characteristics of both groups. For both divers and controls exclusion criteria were (a) presence of neurological, pulmonary, cardiovascular, or metabolic disease, (b) positive test for thrombophilia, and (c) evidence for patent foramen oval. Prior to the beginning of the study, ethical approval was granted From the first author’s university ethics committee. The study obtained ethical permission from the Internal Ethic Review Board – IERB, Department of Education Sciences, Psychology Section, University of Catania (29/11/2018). All participants were informed about the trials of the study and the anonymity of their answers before providing their written consent to participate, in accordance with the Declaration of Helsinki.

**TABLE 1:** Characteristics of study participants.

	**Male divers (*N* = 35)**	**Female divers (*N* = 17)**	**Male controls (*N* = 35)**	**Female controls (*N* = 17)**
Age, years	41.0 ± 6.75	40.9 ± 4.77	38.7 ± 7.51	39.6 ± 6.56
BMI, kg/m^2^	25.7 ± 3.18	25.8 ± 3.11	25.4 ± 2.21	25.6 ± 2.85
Immersions	4881.8 ± 2168.13	3841.5 ± 1060.32		

### Measures

#### Neurological and Neuropsychological Assessment

All participants were tested by the same neuropsychologist (T.M.) using a neuropsychological test battery grouped according to major functional category.

**General mental capability - Wechsler Adult Intelligence Scale– Revised** (WAIS–R; Wechsler, 1981): WAIS–R Full-scale IQ (FSIQ); WAIS–R Verbal IQ (VIQ); WAIS–R Performance IQ (PIQ).

**Executive functioning:** Trail Making Test (Tombaugh, 2004), Trail A and Trail B; Wisconsin Card Sorting Test, WCST (Berg, 1948).

**Reasoning and abstraction- WAIS-R**: Similarities (Sims), Comprehension (Compr). Arithmetic (Arith).

**Visuospatial construction:** Rey Complex Figure (Rey, 1941) scored according to the traditional guidelines developed by Taylor (Spreen and Strauss, 1998): Copy; WAIS-R Block Design, WAIS-R Object Assembly.

**Language:** WAIS-R Vocabulary (Vocab).

**Attention and orientation:** Digit Span-forward and backward (Soylu, 2010), Corsi block-tapping test (Kessels et al., 2000) forward and backward.

**Selective attention capacity and processing speed ability:** Stroop Color and Word Test (Stroop, 1935), score by using the formula originally proposed by Callaway (1959), i.e., time taken to complete the color-word card minus time spent to complete the color card.

**Memory: Wechsler Memory Scale–Revised** (WMS-R; Wechsler, 1987): Immediate Memory (Logical Memory), Delayed Memory (verbal Paired Association) and Working Memory (Letter - Number Sequencing) were evaluated (Axelrod, 2001). Rey Complex Figure: recall.

#### Brain Imaging

Scans were performed with conventional techniques and DTI. Along the anterior/posterior commissure line, conventional axial T1-weighted, T2-weighted, fluid-attenuated inversion recovery (FLAIR), and DTI sequences were acquired with a 1.5T imaging system. Acquisition of DTI images were carried out with a single-shot pulsed-gradient, echo-planar imaging protocol (TR8000 ms, TE109 ms, FOV240 mm, matrix128 × 128, section thickness 3 mm). Diffusion gradients were evaluated in 25 non-collinear directions by using 2 b values (0 and 1500 s/mm^2^). Duration for DTI acquisition was 8 min and 6 s.

**FIGURE 1:**
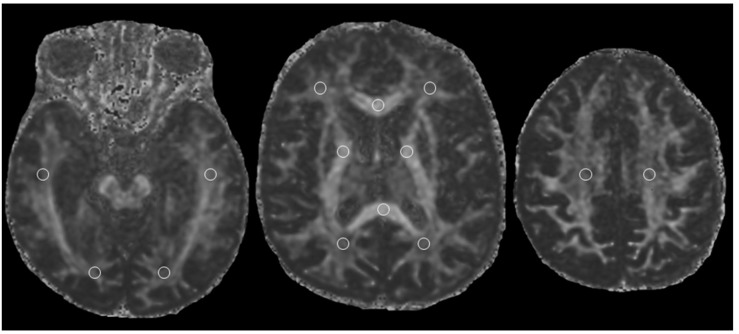
Fractional anisotropy map showing locations white matter Regions of Interest (ROIs).

Oval Regions of Interest (ROIs) were positioned bilaterally on images acquired without diffusion gradients (b 0 s/mm^2^). Small ROIs of 9–16 pixels (31.64–56.25 mm^2^) were located in correspondence of frontal lobe (FL), temporal lobe (TL), parietal lobe (PL), occipital lobe (OL), splenium of corpus callosum (SCC), genu of corpus callosum (GCC), corona radiata (CR), and corticospinal tract (CT) white matter (Figure 1).

For each subject, the measures of FA were separately carried out for each side of every white matter region and then averaged across all the sections bilaterally. FA was calculated using the method proposed by Basser and Pierpaoli (1996) on a pixel-by-pixel basis. The scale of FA ranges from 0 (perfect isotropy) to 1 (perfect anisotropy).

### Statistical Analyses

Differences between the two groups of participants were assessed by one-way analyses of variance (ANOVA) with the nonparametric Kruskal-Wallis test. Voxel-wise group differences in FA were assessed using the ANOVA module in SPM99 followed by group-wise *t*-test comparisons. For these comparisons, significance was determined with a *p*-value of <0.05 (corrected for multiple comparisons at the cluster level) with a seven-voxel extent threshold. Statistical analyses were performed using SPSS v. 23 Statistical Software Package for windows (SPSS Inc., Chicago, IL, United States).

## Results

### Fractional Anisotropy

**FIGURE 2:**
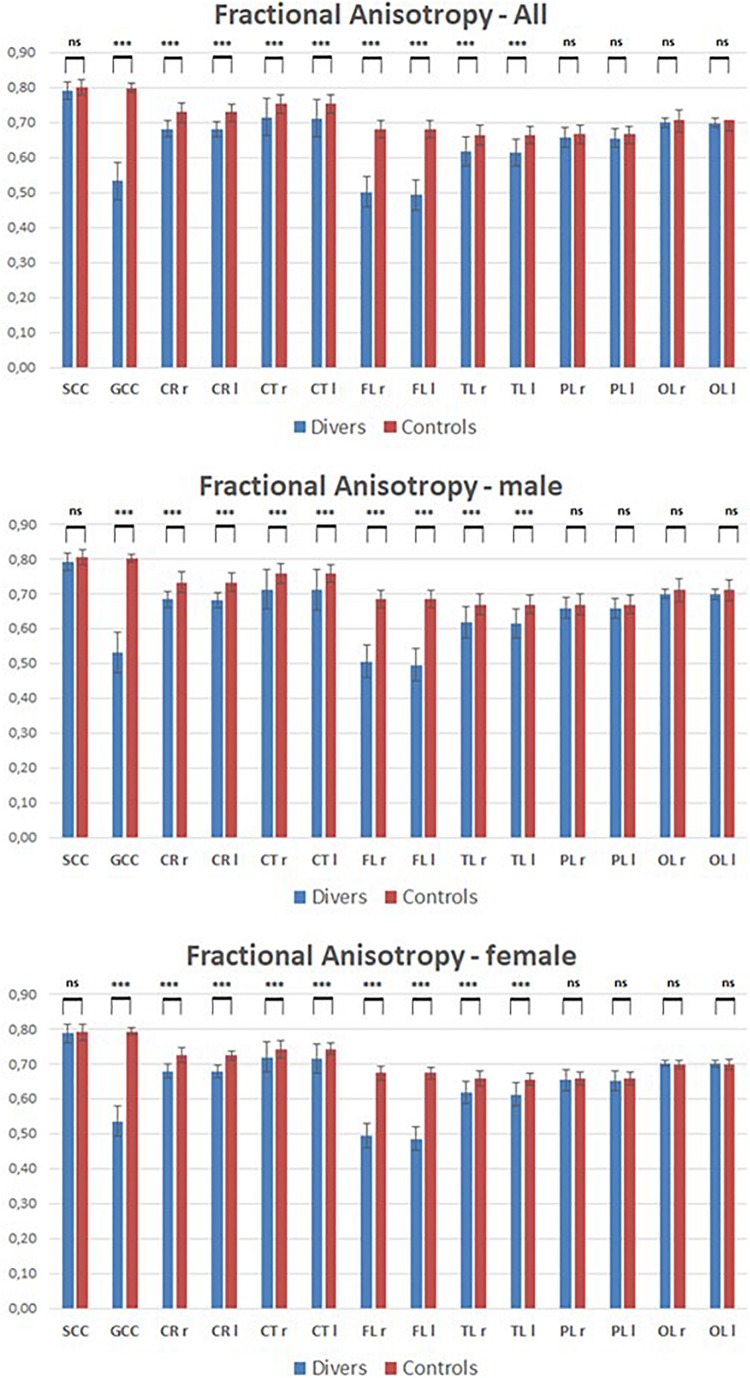
Mean values of Fractional anisotropy measured in scuba divers and controls in correspondence of the 8 ROIs. CR, corona radiata; CT, corticospinal tract; FL, frontal lobe; GCC, genu of corpus callosum; l, left; ns, not significant; OL, occipital lobe; PL, parietal lobe; r, right; SCC, splenium of corpus callosum; TL, temporal lobe. ^∗∗∗^*p* < 0.001.

The MRI of the brains of scuba divers and controls has highlighted the absence of gross lesions. However, as can be seen in Figure 2, if we compared the mean value of FA measured in the 8 ROIs it can be seen that the values measured in correspondence of FL, TL, GCC, CR, and CT were significantly lower in the divers compared to controls. The *post hoc* Dunn’s Multiple Comparison Test has been performed (see Supplementary Table S1). No difference, however, was detected in correspondence of PL, OL, and SCC. The same figure allows detecting how the same pattern found on the entire sample was observed, without statistically significant differences, both in women and in men. Moreover, the figure shows that it was not possible to highlight statistically significant differences, for the different ROIs, between right and left side.

**FIGURE 3:**
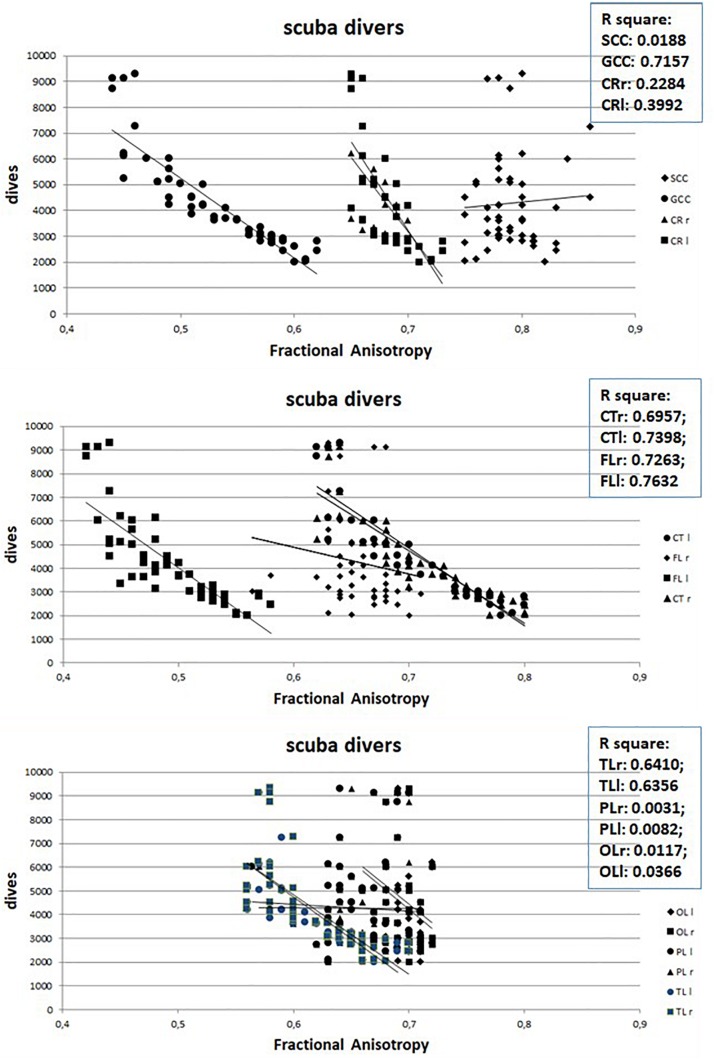
Relation between Fractional Anisotropy and number of dives of divers. Abbreviations as in Figure 2.

However, when in scuba divers the mean value of the FA, measured in the different ROIs, is correlated with the number of dives carried out by each of them, a strong negative correlation emerges, as can be seen in Figure 3, with the exclusion of SCC as well as of left and right PL and OL. Nor were any gender-dependent differences found.

**FIGURE 4:**
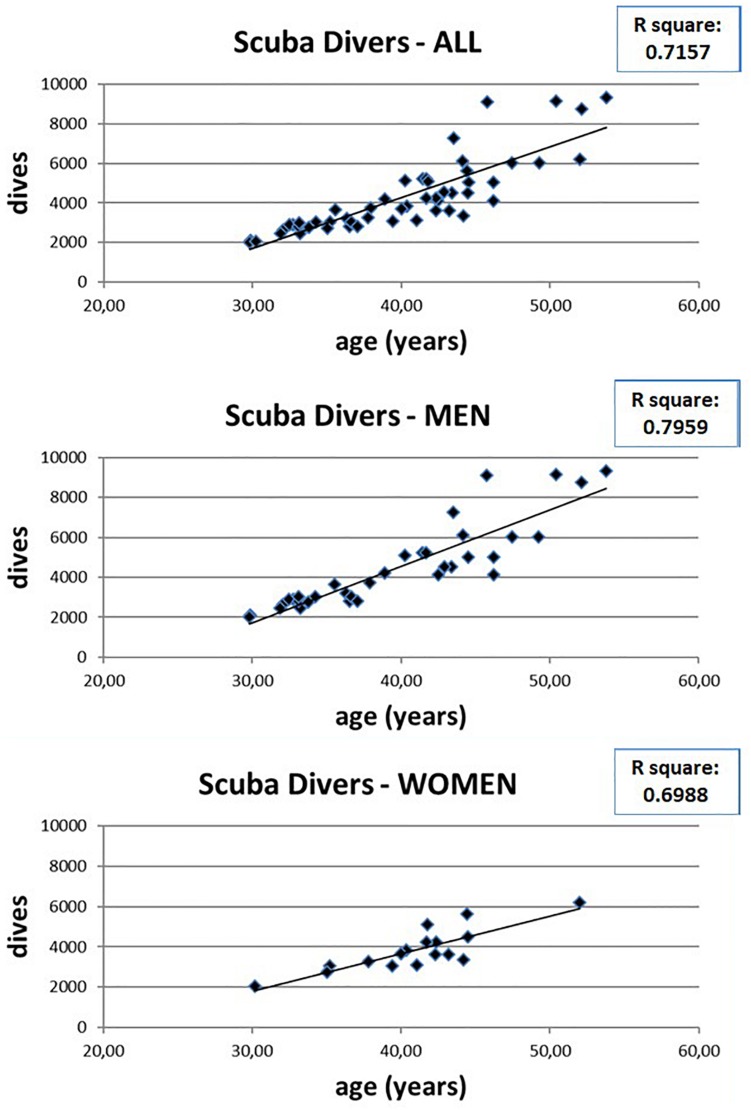
Relation between age and number of dives in scuba divers, as well as in men and women separately.

**FIGURE 5:**
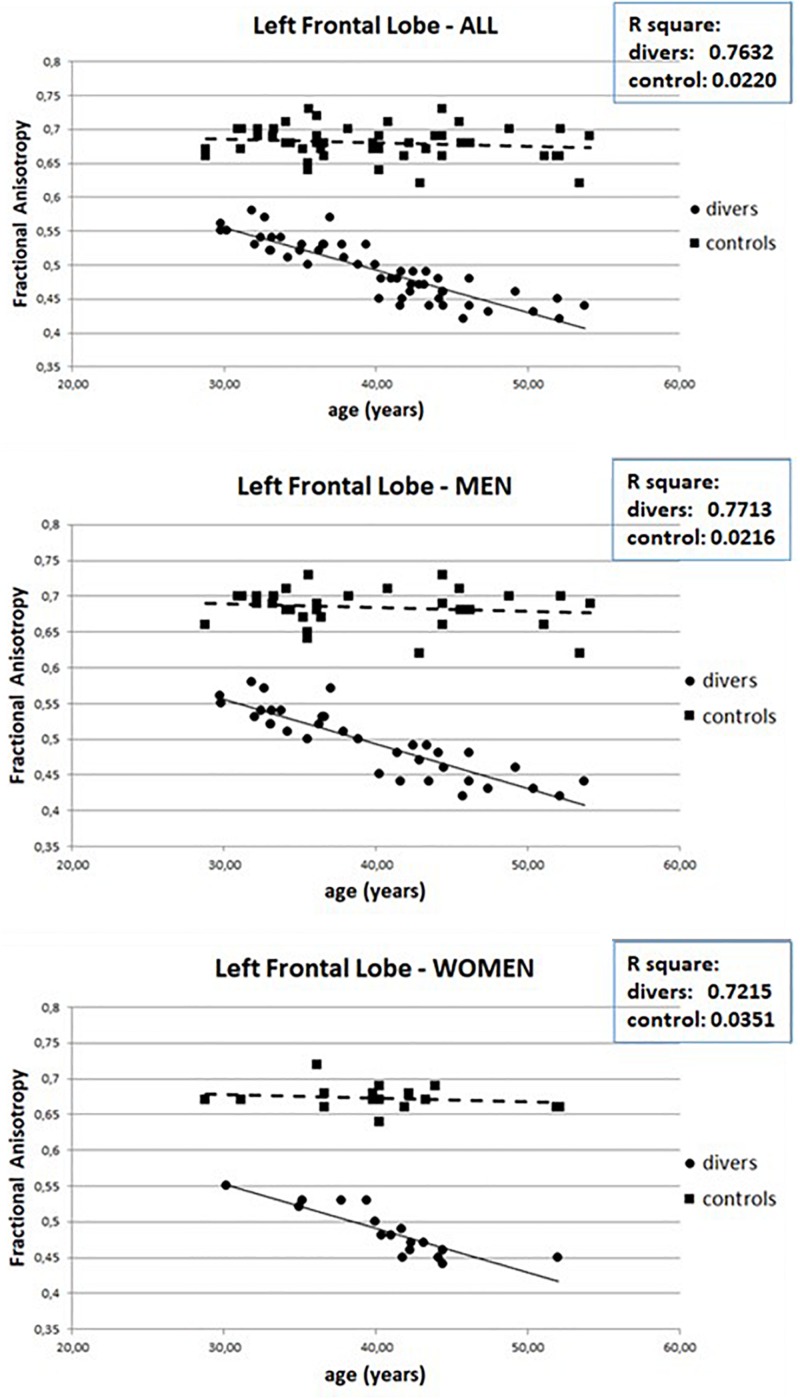
Relation between Fractional Anisotropy and age in scuba divers and controls in left FL.

However, the number of dives obviously increases with increasing age (Figure 4) so it is possible that the observed WM anomalies could depend on age and not on the number of dives. To evaluate this possibility, we connected the values of FA with the age of both scuba divers as well as healthy controls. However, as can be seen in Figure 5 where data relating to the left FL are shown, while in scuba divers the values of FA were significantly related with age, the healthy controls did not exhibit any statistically significant correlation. Statistically significant relations were also obtained by relating with age of divers FA values of right FL, GCC, as well as CT and TL of both sides; no relationship between age and FA was detected in controls. Therefore, it is possible to conclude that the changes of FA observed in scuba divers are related to the number of dives and not to age.

### Neuropsychological Testing

**FIGURE 6:**
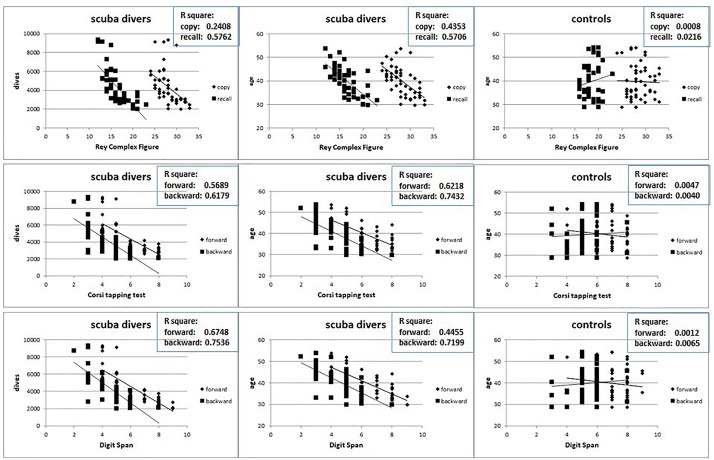
Relation between the results at Ray Complex Figure **(top row)**, Corsi tapping test **(middle row)** and Digit Span **(bottom row)** and the number of dives and age in divers **(left and central columns)** and age in controls **(right columns)**.

Data from neuropsychological testing reveal small but significant impaired performances that increases with the number of dives. In Figure 6 the results obtained for the Ray Complex Figure (top row), for the Corsi tapping test (middle row) and for the Digit Span (bottom row) are reported. The left column shows the relationships between the results obtained in the test and the dives numbers, the center column shows the relationships between the results obtained in the test and the age of the divers, while the right column shows the relationships between the results obtained in the test and the age of the controls.

It is possible to detect that, while in scuba divers there is a significant negative relation between the test’s results and both the number of dives and the age, no relation has been highlighted between the test’s results and age in the controls. Also in this case, no gender differences were detected (data not shown).

**FIGURE 7:**
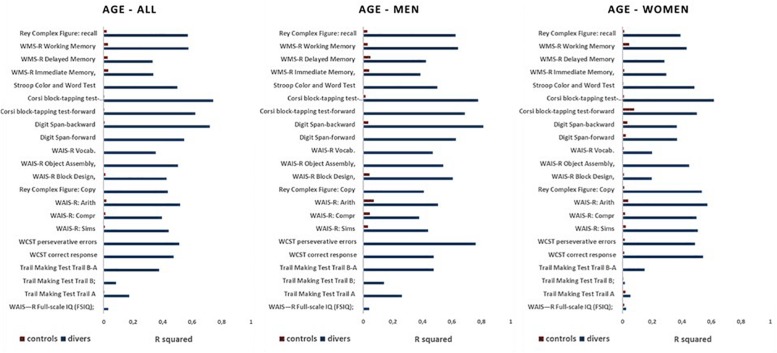
Values of *R* square measured for relations between the results at all neuropsychological tests and the age in divers (blue) and controls (red) in the whole sample (all), as well as in men and women separately.

Figure 7 shows the R square values obtained from the correlations, both in scuba divers and controls, between the age of the subjects and the scores obtained in the different neuropsychological tests administered.

As can be seen, in all scuba divers the scores of all the different neuropsychological tests showed correlations with the age of the subjects with R square values that were less than 0.3 only for FSIQ, Trail Making Test Trail A and Trail Making Test Trail B. In the controls, however, there is no correlation between scores in neuropsychological tests and the age of the subjects, with values of *R* square always below 0.1. Nor were any gender-dependent differences found.

## Discussion

In recent years a number of studies have highlighted the presence of mild but significant cognitive deficits in scuba divers that have performed a high number of dives (Kowalski et al., 2011; Hemelryck et al., 2014; Ergen et al., 2017; Howle et al., 2017; Bosco et al., 2018; Verratti et al., 2019).

The present study aimed to assess whether underwater diving and compressed air work is capable to induce, in a dose-dependent manner, alterations of the cerebral WM and, consequently, to produce alterations of cognitive functions.

The results showed alterations of the WM both in terms of changes in FA; these variations, statistically significant but quantitatively quite modest, were mainly observed in the WM of the anterior part of the brain; no differences were observed between left and right hemisphere and between women and men.

The alterations of WM were positively related to the number of dives and the age of the divers. In controls, on the other hand, no correlation was found between the FA values of the WM and the age of the participants. This led to the conclusion that the changes of FA are more likely a result of the number of dives and are not due to the possibility that more experienced divers are too likely to be older. The absence of changes in the WM of controls is likely to be dependent on the relatively young age of the subjects (mean value 39.7 years ±7.22 SD), none of which reached 55 years (Chen et al., 2019).

In divers, the alterations of the WM were associated with changes, also in this case statistically significant but quantitatively quite modest, of the cognitive functions, in particular of those dependent on the prefrontal cortex (Coco et al., 2009; Ergen et al., 2017). The results of the present study are in agreement with cognitive deficits previously described in professional scuba divers. In particular, in this research cognitive deficits were found mainly, but not exclusively, in the domain of frontal functions. Kowalski et al. (2011) found delays in reaction time in 25 very experienced divers, compared to 23 non-divers. The findings showed that a lasting cerebral damage possibly develops only after a long diving career consisting of numerous decompressions, and repetitive dives. In addition, their results of the controls suggest that age had no direct influence on reaction times. Investigating long-term effects of diving on cognitive function in 44 experienced scuba divers, Hemelryck et al. (2014) found a worse performance in short-term memory, compared to controls.

The present study leads to the conclusion that repeated dives, even performed in compliance with the current decompression tables, could progressively induce the formation of microbubbles in the myelin, with the result of micro lesions in the axonal sheet capable of altering the functioning of the neuron. Several mathematical models to predict the probability of bubble formation for a given dive profile were proposed; the algorithms constructed on these models are the basis of decompression tables (Parker et al., 1992). Modern dive computers can evaluate in real-time the decompression status and report it to the diver (Lang and Hamilton, 1989). The present results suggest that these mathematical models are able to calculate the formation of bubbles in a short-medium temporal dimension, as in the case of divers who in their life do some hundreds of dives. However, evidently these models cannot predict the slow formation of microbubbles over thousands of dives. In conclusion, this study has shown that in individuals who have made at least two thousand scuba diving with compressed air can be seen modification of the cerebral WM in a dose-dependent manner. These alterations were particularly evident in the FL and in the GCC, without differences of side or gender. It has also been possible to detect that these structural modifications were associated with small but significant cognitive alterations that mainly concerned the executive functions, without gender differences.

Despite the gender differences in diver performance still being debated, Brebeck et al. (2017) demonstrated that female divers performed better than male divers on the memory tests, and Pauls et al. (2013) found highest gender differences in non-visuospatial abilities with an advantage for women.

In conclusion, it can be determined that after the diving cognitive response amount is low and it’s better that divers instantly refrain from doing something with elevated attention after diving.

Of course, this study has certain limitations. For one, a weakness could be represented by the heterogeneous distribution over the years of their dives. However, these factors will make the achievement of statistically significant differences less likely. Given this heterogeneity, great care was taken to ensure randomization to the control group and experimental groups. Furthermore, in light of the sparse data on female divers, our findings suggest that gender differences in diver performance may merit further investigation.

## Data Availability Statement

The datasets generated for this study are available on request to the corresponding author.

## Ethics Statement

This study was carried out in accordance with the recommendations of Ethical Committees of our Institutions with written informed consent from all subjects. All subjects gave written informed consent in accordance with the 1964 Declaration of Helsinki. The protocol obtained ethical permission from the Internal Ethic Review Board – IERB, Department of Education Sciences, Psychology Section, University of Catania (29/11/2018).

## Author Contributions

MC, ViP, AB, and VaP contributed to the conception and design of the study. TM was responsible for neuropsychological testing whereas GG and AS performed the Fractional anisotropy evaluation. MC, AB, DD, and ViP were responsible for data collection and statistical analysis. MC, AB, and ViP was responsible for drafting and finalization of the manuscript. All authors contributed to the manuscript revision and approved the submitted version of the manuscript.

## Conflict of Interest

The authors declare that the research was conducted in the absence of any commercial or financial relationships that could be construed as a potential conflict of interest.
