# Supplementary material for: Cognitive Deficits and White Matter Alterations in Highly Trained Scuba Divers
Source: Front Psychol. 2019 Oct 22;10:2376. doi: 10.3389/fpsyg.2019.02376 (PMC6817599; doi:10.3389/fpsyg.2019.02376)
Supplement: Supplementary file 1 [file Table_2.pdf]

Dunn's multiple comparisons test	Mean rank diff,	Summary
SCC vs. GCC	390,9	****
SCC vs. CR r	164,6	***
SCC vs. CR l	168,3	****
SCC vs. CT r	117,7	*
SCC vs. CT l	123,9	*
SCC vs. FL r	412,7	****
SCC vs. FL l	419,7	****
SCC vs. TL r	295,1	****
SCC vs. TL l	303,4	****
SCC vs. PL r	226,5	****
SCC vs. PL l	233,0	****
SCC vs. OL r	112,4	ns
SCC vs. OL l	115,5	ns
GCC vs. CR r	-226,3	****
GCC vs. CR l	-222,6	****
GCC vs. CT r	-273,2	****
GCC vs. CT l	-267,1	****
GCC vs. FL r	21,77	ns
GCC vs. FL l	28,76	ns
GCC vs. TL r	-95,87	ns
GCC vs. TL l	-87,50	ns
GCC vs. PL r	-164,4	***
GCC vs. PL l	-158,0	***
GCC vs. OL r	-278,6	****
GCC vs. OL l	-275,4	****
CR r vs. CR l	3,700	ns
CR r vs. CT r	-46,93	ns
CR r vs. CT l	-40,74	ns
CR r vs. FL r	248,1	****
CR r vs. FL l	255,1	****
CR r vs. TL r	130,4	*
CR r vs. TL l	138,8	**
CR r vs. PL r	61,90	ns
CR r vs. PL l	68,33	ns
CR r vs. OL r	-52,27	ns
CR r vs. OL l	-49,09	ns
CR l vs. CT r	-50,63	ns
CR l vs. CT l	-44,44	ns
CR l vs. FL r	244,4	****
CR l vs. FL l	251,4	****
CR l vs. TL r	126,7	*
CR l vs. TL l	135,1	**
CR l vs. PL r	58,20	ns
CR l vs. PL l	64,63	ns
CR l vs. OL r	-55,97	ns
CR l vs. OL l	-52,79	ns
CT r vs. CT l	6,186	ns
CT r vs. FL r	295,0	****
CT r vs. FL l	302,0	****
CT r vs. TL r	177,4	****
CT r vs. TL l	185,7	****
CT r vs. PL r	108,8	ns
CT r vs. PL l	115,3	ns
CT r vs. OL r	-5,343	ns
CT r vs. OL l	-2,157	ns
CT l vs. FL r	288,8	****
CT l vs. FL l	295,8	****
CT l vs. TL r	171,2	****
CT l vs. TL l	179,6	****
CT l vs. PL r	102,6	ns
CT l vs. PL l	109,1	ns
CT l vs. OL r	-11,53	ns
CT l vs. OL l	-8,343	ns
FL r vs. FL l	6,986	ns
FL r vs. TL r	-117,6	*
FL r vs. TL l	-109,3	ns
FL r vs. PL r	-186,2	****
FL r vs. PL l	-179,8	****
FL r vs. OL r	-300,4	****
FL r vs. OL l	-297,2	****
FL l vs. TL r	-124,6	*
FL l vs. TL l	-116,3	ns
FL l vs. PL r	-193,2	****
FL l vs. PL l	-186,7	****
FL l vs. OL r	-307,3	****
FL l vs. OL l	-304,2	****
TL r vs. TL l	8,371	ns
TL r vs. PL r	-68,54	ns
TL r vs. PL l	-62,11	ns
TL r vs. OL r	-182,7	****
TL r vs. OL l	-179,5	****
TL l vs. PL r	-76,91	ns
TL l vs. PL l	-70,49	ns
TL l vs. OL r	-191,1	****
TL l vs. OL l	-187,9	****
PL r vs. PL l	6,429	ns
PL r vs. OL r	-114,2	ns
PL r vs. OL l	-111,0	ns
PL l vs. OL r	-120,6	*
PL l vs. OL l	-117,4	*
OL r vs. OL l	3,186	ns

Supplementary Table S1. Results of post hoc Dunn's Multiple Comparison Test for the entire sample of scuba divers. Abbreviations: CR, corona radiata; CT, corticospinal tract; FL, frontal lobe; GCC, genu of corpus callosum; l, left; ns, not significant; OL, occipital lobe; PL, parietal lobe; r, right; SCC, splenium of corpus callosum; TL, temporal lobe. ****, $p < 0.0001$; ***, $p < 0.001$; **, $p < 0.01$; *, $p < 0.05$.
